# Single administration of vildagliptin attenuates postprandial hypertriglyceridemia and endothelial dysfunction in normoglycemic individuals

**DOI:** 10.3892/etm.2014.2051

**Published:** 2014-11-05

**Authors:** KAORU NOGUCHI, MINORU HIROTA, TORU MIYOSHI, YOSHINORI TANI, YOKO NODA, HIROSHI ITO, SEIJI NANBA

**Affiliations:** 1 Department of Cardiology, Okayama Rosai Hospital, Okayama 702-8055, Japan; 2 Department of Cardiovascular Medicine, Okayama University Graduate School of Medicine, Dentistry and Pharmaceutical Sciences, Okayama 700-8558, Japan

**Keywords:** endothelial function, postprandial lipemia, dipeptidyl peptidase 4

## Abstract

Postprandial hypertriglyceridemia impairs endothelial function and plays an important role in the development of atherosclerosis. The aim of the present study was to examine the postprandial effects of the dipeptidyl peptidase-4 inhibitor vildagliptin and the α-glucosidase inhibitor voglibose on endothelial dysfunction and lipid profiles following a single administration. A randomized cross-over trial using 11 normoglycemic individuals was performed. The postprandial effects of a single administration of vildagliptin (50 mg) or voglibose (0.3 mg) on endothelial function were analyzed using brachial artery flow-mediated dilation (FMD) and lipid profiles during fasting and 1.5 and 3 h after an oral cookie-loading test. Compared with voglibose, vildagliptin significantly suppressed postprandial endothelial dysfunction, (%FMD, −1.6±0.9 vildagliptin vs. −4.0±0.7 voglibose; P=0.01) and the postprandial incremental increase in the triglyceride level (28±18 vildagliptin vs. 51±26 mg/dl voglibose; P=0.01) 3 h after a cookie-loading test. In addition, vildagliptin significantly increased the levels of glucagon-like peptide-1 compared with voglibose 3 h after a loading cookie test (4.4±0.6 vs. 2.9±0.7 pmol/l, respectively; P=0.04). No significant differences in the levels of glucose, apolipoprotein B-48, glucagon or insulin were observed between vildagliptin and voglibose treatments. In conclusion, a single administration of vildagliptin attenuated postprandial endothelial dysfunction and postprandial hypertriglyceridemia, suggesting that vildagliptin may be a promising antiatherogenic agent.

## Introduction

Non-fasting postprandial triglyceride (TG) concentrations are more effective at predicting cardiovascular risk than fasting TG concentrations; this association is independent of traditional coronary risk factors (1,2). TG-rich lipoproteins, including chylomicrons assembled by TGs, dietary cholesterol and apolipoprotein B-48 (apoB-48), are extremely atherogenic and contribute to coronary heart disease development (3). Even in normolipidemic individuals, postprandial hypertriglyceridemia contributes to the generation of proinflammatory cytokines and oxidative stress, causing endothelial dysfunction (4,5). Thus, the identification of novel therapeutic approaches to target postprandial lipid concentrations is of significant interest.

Vildagliptin is a selective inhibitor of dipeptidyl peptidase-4 (DPP4) and has been demonstrated to reduce the fasting and postprandial glucose levels in patients with type 2 diabetes, possibly by inhibiting the inactivation of glucagon-like peptide-1 (GLP-1) (6–8). Clinical studies have revealed that the long-term administration of DPP4 inhibitors improves the postprandial atherogenic levels of TG-rich lipoprotein and/or endothelial dysfunction in patients with type 2 diabetes and healthy volunteers (9–11). However, Ayaori *et al* reported that the effects of other DPP4 inhibitors attenuated endothelial dysfunction in patients with type 2 diabetes (12). Thus, the difference in effects on endothelial function among DPP4 inhibitors remains controversial. Furthermore, following long-term treatment with DPP4 inhibitors, the discrimination between the acute and chronic effects of vildagliptin is difficult.

α-glucosidase inhibitors, which lower postprandial plasma glucose levels by delaying the absorption of sugars from the digestive system, are used widely in the treatment of type 2 diabetes. A previous study also showed that treatment with voglibose improves endothelial function in type diabetes mellitus (13). Another study investigated the effect of voglibose on postprandial triglyceridemia following a meal test, but failed to show the benefit of voglibose compared with the placebo (14). The aim of the present study was to examine the postprandial effects of a single administration of vildagliptin and voglibose, an α-glucosidase inhibitor, on endothelial dysfunction and the lipid profile in normal individuals.

## Materials and methods

### Participants and study design

**Figure 1:**
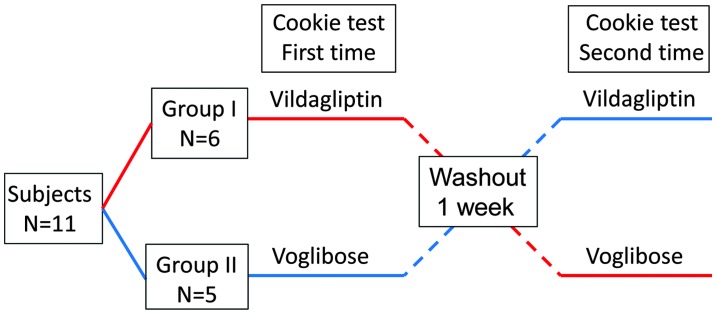
Study design. The present study was a randomized, double-blind, cross-over design. Prior to each cookie-loading test, 50 mg vildagliptin or 0.3 mg voglibose were administered in a blinded manner.

The procedures carried out in the present study were approved by the Ethics Committee of Okayama Rohsai Hospital (Okayama, Japan) and written informed consent was obtained from all volunteers prior to the study commencement. The study procedures conformed to the ethical guidelines of the 1975 Declaration of Helsinki, as reflected by the *a priori* approval of the Ethics Committee of the hospital. The study was a randomized, double-blind, cross-over study (Fig. 1), and all procedures were carried out at the Okayama Rohsai Hospital. A total of 11 volunteers were recruited through clinical study sites and community outreach between September and November 2012. A single dose of vildagliptin (50 mg; Novartis Pharmaceuticals Co., Tokyo, Japan) or voglibose (0.3 mg; Takeda Pharmaceutical Co Ltd, Tokyo, Japan) was administered and a one-week washout period was implemented prior to the administration of the second drug. The drugs were administered in a random order. All volunteers underwent medical check-ups and family histories were obtained in medical interviews. None of the 11 participants had hypertension, impaired glucose tolerance, dyslipidemia, or cerebrovascular or cardiovascular disease. The pre-specified primary outcome measure was the difference in the decrease in brachial artery flow-mediated dilation (FMD) following the cookie-loading test, and the secondary outcome measure was the difference in lipids profiles following the cookie-loading test.

### Cookie-loading test procedure

Following overnight fasting for ≥8–12 h, one tablet of vildagliptin or voglibose was taken 1 h prior to the cookie-loading test. The cookie consisted of 75 g carbohydrate, 28.5 g fat and 8 g protein with a total of 592 kcal per carton (Saraya Co., Osaka, Japan). Participants were instructed to consume the cookie with water within 20 min. Time measurements were initiated when half the cookie had been consumed. Biological parameters in the blood and endothelium-dependent FMD were assessed during fasting and 1.5 and 3 h after the cookie-loading test.

### Measurement of biochemical parameters

The following parameters were measured in the blood by SRL Inc., (Tokyo, Japan) in the state of fasting prior to cookie ingestion: Serum total cholesterol (total-C), TG, low-density lipoprotein cholesterol (LDL-C), high-density lipoprotein cholesterol (HDL-C), apoB-48, glucagon, GLP-1, insulin, hemoglobin A1c and glucose levels. Serum levels of total-C, TG, LDL-C, apoB-48, GLP-1, glucagon, insulin and glucose were measured 1.5 and 3 h after the cookie-loading test.

### Measurement of FMD

During fasting and 1.5 and 3 h after cookie ingestion, measurements of FMD were performed by the same technician who was blinded to the study design and medication status. FMD was assessed as a parameter of vasodilation according to the guidelines for ultrasound assessment of FMD of the brachial artery using a 10-MHz linear array transducer probe (Unex Co., Nagoya, Japan) (11). Longitudinal images of the brachial artery at baseline were recorded with a stereotactic arm and the artery diameter was measured following supine rest for ≥5 min. The diameter of the artery was measured from the clear anterior (media-adventitia) and posterior (intima-media) interfaces, which were manually determined. Suprasystolic compression (50 mmHg higher than the systolic blood pressure) was subsequently performed on the right forearm for 5 min and measurements of the artery diameter were made continuously from 30 sec before to ≥2 min after cuff release. Maximum vasodilation was evaluated from the change in artery diameter following release from occlusion (%FMD).

### Statistical analysis

The sample size was determined based on the estimated FMD reported in a recent study (11). It was assumed that the mean improvement in postprandial %FMD was 2.7% and that the standard deviation was 2.0%. To use a two-sided test for differences, a minimal sample size of 10 individuals was required in each group to detect statistical differences in %FMD with a power of 80% and an α-type error of 5%. Results are expressed as the mean ± standard error. Differences in lipid profile and endothelial function between the two groups were compared using the Wilcoxon signed-rank test. P<0.05 was considered to indicate a statistically significant difference.

## Results

A total of 11 volunteers (seven males and four females) were recruited. The mean age and body mass index of the volunteers was 32±2 years and 22.0±0.7 kg/m^2^, respectively. Participants maintained their weight throughout the study period.

**Figure 2:**
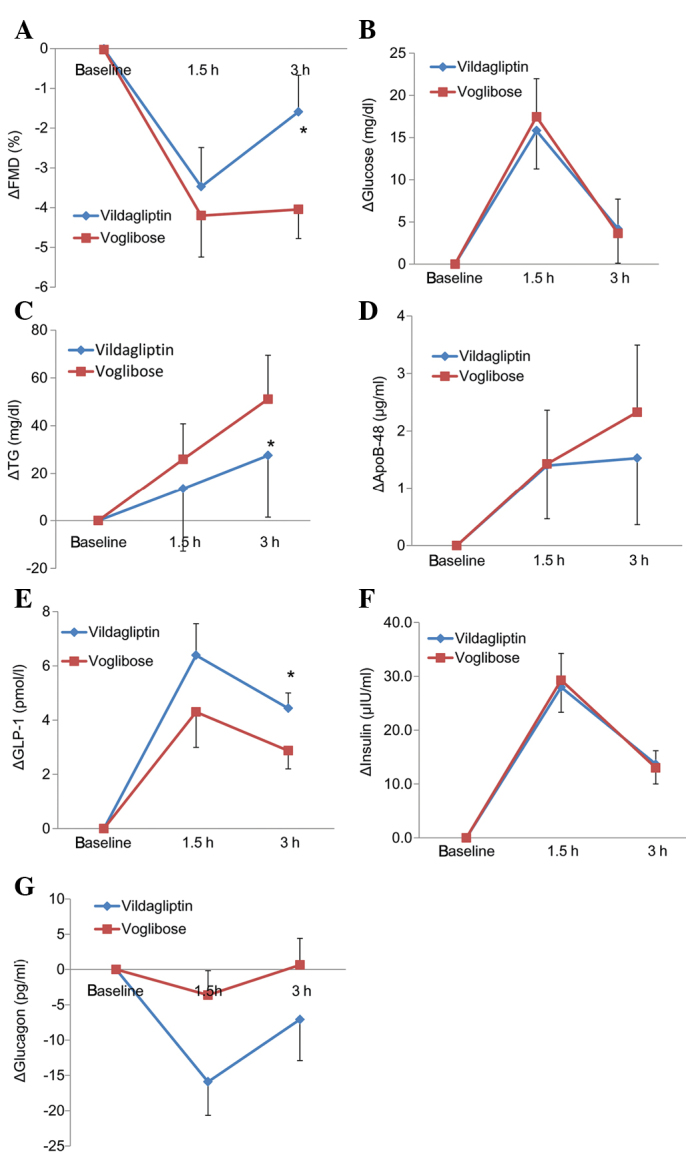
Effects of vildagliptin or voglibose treatment on changes in postprandial levels of (A) FMD, (B) glucose, (C) TG, (D) apoB-48, (E) GLP-1, (F) insulin and (G) glucagon in 11 individuals. Data are presented as the mean ± standard error. FMD, flow-mediated dilation; TG, triglyceride; apoB-48, apolipoprotein B-48; GLP-1, glucagon-like peptide-1.

The comparison of postprandial endothelial function, which was assessed as the %FMD, between the vildagliptin and voglibose groups is shown in Table I. In the voglibose group, the postprandial %FMD decreased significantly until 3 h; however, the decrease in postprandial %FMD in the vildagliptin group was higher compared with that in the voglibose group (9.7±0.9 vs. 7.2±0.7%, respectively; Table I). The changes in the %FMD are shown in Table I and Fig. 2A. The change in the %FMD from baseline to 3 h was significantly lower in the vildagliptin group compared with that in the voglibose group (−1.6±0.9 vs. −4.0±0.7%, respectively; P=0.01; Fig. 2A).

The levels of lipid/lipoprotein, glucose, glucagon, GLP-1 and insulin in the postprandial state are shown in Table I and Fig. 2. The level of apoB-48 1.5 h after the cookie-loading test in the vildagliptin group was significantly lower compared with that in the voglibose group. The levels of GLP-1 1.5 and 3 h after cookie ingestion in the vildagliptin group were significantly higher than those in the voglibose group. Changes in the levels of lipid/lipoprotein, glucose, glucagon, GLP-1 and insulin following the cookie-loading test are shown in Fig. 2 and Table I. The change in the level of TG from baseline to 3 h was significantly lower in the vildagliptin group compared with that in the voglibose group. The change in the level of GLP-1 from baseline to 3 h was significantly higher in the vildagliptin group than that in the voglibose group. The changes in the levels of apoB-48, glucose, glucagon and insulin from baseline to 3 h did not significantly differ between the vildagliptin and voglibose groups.

## Discussion

The results of the present study demonstrated that a single administration of vildagliptin significantly attenuated postprandial endothelial dysfunction (which was induced by a standard meal loading test) when compared with voglibose and that vildagliptin reduced postprandial levels of TG.

Our previous study revealed that alogliptin treatment for one week ameliorated postprandial hypertriglyceridemia and endothelial dysfunction in non-diabetic individuals (11). Other studies have demonstrated that treatment with vildagliptin for four weeks and sitagliptin for six weeks decreased the postprandial levels of TG and apoB-48 following a fat-loading test in patients with type 2 diabetes (9,10). However, the present study is, to the best of our knowledge, the first to demonstrate that a single administration of vildagliptin reduces the postprandial increase in TG that accompanies postprandial endothelial dysfunction in healthy individuals. Following long-term treatment with DPP4 inhibitors, the discrimination between the acute and chronic effects of vildagliptin is difficult. The present study clearly demonstrated the acute effects of vildagliptin on postprandial endothelial dysfunction and hypertriglyceridemia.

There are several possible mechanisms underlying the effect of vildagliptin on postprandial endothelial dysfunction and hypertriglyceridemia. Regarding the attenuation of endothelial dysfunction, the increased expression of GLP-1 induced by vildagliptin may directly protect endothelial cells from hypertriglyceridemia. Clinical studies have demonstrated that GLP-1 analogs improve endothelial function in patients with type 2 diabetes (15–17). An experimental study reported that GLP-1 activated endothelial nitric oxide synthase in endothelial cells (18). Regarding the attenuation of hypertriglyceridemia, one study demonstrated that GLP-1 affected intestinal TG absorption (19), possibly by inhibiting gastric lipase (20). Another study has shown that GLP-1 may directly regulate lipoprotein assembly or the secretion of chylomicron in enterocytes (21).

A number of previous studies have demonstrated that treatment with α-glucosidase inhibitors, including acarbose or miglitol, for >3 months improves postprandial endothelial dysfunction in patients with diabetes or coronary artery disease (22,23). However, only one study reported that a single administration of miglitol significantly improved postprandial glucose and lipid metabolism and incretin secretion, and attenuated endothelial dysfunction in patients with diabetes following a standard meal loading; no favorable effects were observed following the administration of voglibose or a placebo (14). Despite the fact that the present study lacked a placebo group, the previous results support the findings of the current study that a single administration of vildagliptin significantly reduced the increase in TG following the cookie-loading test.

The present study had several important limitations. Firstly, the number of participants was small and therefore selection bias may have occurred. Secondly, no standardized method for assessing postprandial hypertriglyceridemia has been established. Thus, a range of fat-loading tests, including an oral fat meal, fat cream intake and intravenous fat loading, have been used in previous studies (14,24). The present study utilized the cookie-loading test, which provided sufficient information regarding glucose intolerance and postprandial hypertriglyceridemia (11). Although the cookie provided a fixed amount of fat (28.5 g) per individual, the amount of fat administered per body surface area was not regulated. Therefore, the contribution of the fat metabolism of each individual cannot be excluded as an influential factor.

In conclusion, the present study compared the postprandial effects of vildagliptin and voglibose on endothelial dysfunction and lipid profiles. It was demonstrated that a single administration of the DPP4 inhibitor vildagliptin was effective at ameliorating the postprandial endothelial dysfunction, accompanied by a reduction in the postprandial elevation of TG. Vildagliptin shows potential as a drug to target cardiovascular disease by ameliorating endothelial dysfunction in the postprandial state, even in low-risk individuals.

## Figures and Tables

**Table I:** Lipid profile, apolipoprotein B-48, glucose, glucagon, GLP-1, insulin and %FMD measurements after the cookie test.

A, Biochemical parameters			

Parameter	Fasting	1.5 h	3 h
Total-cholesterol (mg/dl)			
Voglibose	188±11	180±11	175±15
Vildagliptin	190±13	192±13	187±12
LDL-cholesterol (mg/dl)			
Voglibose	114±11	107±10	110±10
Vildagliptin	110±13	113±14	107±12
HDL-cholesterol (mg/dl)			
Voglibose	58±3	57±3	56±3
Vildagliptin	60±3	60±3	59±3
Triglyceride (mg/dl)			
Voglibose	98±17	124±26	149±26
Vildagliptin	94±15	108±15	122±18
Apolipoprotein B-48 (μg/ml)			
Voglibose	4.9±1.0	6.3±0.9	7.2±1.2
Vildagliptin	4.0±0.6	5.4±0.7^a^	5.5±0.7
Glucose (mg/dl)			
Voglibose	98±2	115±6	101±4
Vildagliptin	97±2	113±5	101±5
Glucagon (pg/ml)			
Voglibose	71±8	68±9	72±9
Vildagliptin	76±8	61±8	69±9
GLP-1 (pmol/l)			
Voglibose	3.4±0.7	7.7±1.5	6.3±1.1
Vildagliptin	4.6±0.9	11.0±1.7^a^	9.0±1.1^a^
Insulin (μU/ml)			
Voglibose	5.2±0.7	34.5±5.2	18.2±3.5
Vildagliptin	6.3±0.9	34.3±5.4	20.0±4.3

B, Endothelial function			

Parameter	Fasting	1.5 h	3 h

FMD (%)			
Voglibose	11.2±1.2	7.0±1.0	7.2±0.7^a^
Vildagliptin	11.3±1.3	7.8±1.0	9.7±0.9

Data are presented as the mean ± standard error or frequency count (percentages), as appropriate.

a P<0.05 vs. the voglibose group.

LDL, low-density lipoprotein; HDL, high-density lipoprotein; GLP-1, glucagon-like peptide-1; FMD, flow-mediated dilation.

## References

[1] Bansal S, Buring JE, Rifai N (2007). Fasting compared with nonfasting triglycerides and risk of cardiovascular events in women. JAMA.

[2] Nordestgaard BG, Benn M, Schnohr P, Tybjaerg-Hansen A (2007). Nonfasting triglycerides and risk of myocardial infarction, ischemic heart disease, and death in men and women. JAMA.

[3] Véniant MM, Pierotti V, Newland D (1997). Susceptibility to atherosclerosis in mice expressing exclusively apolipoprotein B48 or apolipoprotein B100. J Clin Invest.

[4] Bae JH, Bassenge E, Kim KB (2001). Postprandial hypertriglyceridemia impairs endothelial function by enhanced oxidant stress. Atherosclerosis.

[5] van Oostrom AJ, Sijmonsma TP, Verseyden C (2003). Postprandial recruitment of neutrophils may contribute to endothelial dysfunction. J Lipid Res.

[6] DeFronzo RA, Fleck PR, Wilson CA, Mekki Q (2008). Alogliptin Study 010 Group: Efficacy and safety of the dipeptidyl peptidase-4 inhibitor alogliptin in patients with type 2 diabetes and inadequate glycemic control: a randomized, double-blind, placebo-controlled study. Diabetes Care.

[7] Feng J, Zhang Z, Wallace MB (2007). Discovery of alogliptin: a potent, selective, bioavailable, and efficacious inhibitor of dipeptidyl peptidase IV. J Med Chem.

[8] Moritoh Y, Takeuchi K, Asakawa T, Kataoka O, Odaka H (2008). Chronic administration of alogliptin, a novel, potent, and highly selective dipeptidyl peptidase-4 inhibitor, improves glycemic control and beta-cell function in obese diabetic ob/ob mice. Eur J Pharmacol.

[9] Matikainen N, Mänttäri S, Schweizer A (2006). Vildagliptin therapy reduces postprandial intestinal triglyceride-rich lipoprotein particles in patients with type 2 diabetes. Diabetologia.

[10] Tremblay AJ, Lamarche B, Deacon CF, Weisnagel SJ, Couture P (2011). Effect of sitagliptin therapy on postprandial lipoprotein levels in patients with type 2 diabetes. Diabetes Obes Metab.

[11] Noda Y, Miyoshi T, Oe H (2013). Alogliptin ameliorates postprandial lipemia and postprandial endothelial dysfunction in non-diabetic subjects: a preliminary report. Cardiovasc Diabetol.

[12] Ayaori M, Iwakami N, Uto-Kondo H (2013). Dipeptidyl peptidase-4 inhibitors attenuate endothelial function as evaluated by flow-mediated vasodilatation in type 2 diabetic patients. J Am Heart Assoc.

[13] Nakamura K, Oe H, Kihara H (2014). DPP-4 inhibitor and alpha-glucosidase inhibitor equally improve endothelial function in patients with type 2 diabetes: EDGE study. Cardiovasc Diabetol.

[14] Hiki M, Shimada K, Kiyanagi T (2010). Single administration of alpha-glucosidase inhibitors on endothelial function and incretin secretion in diabetic patients with coronary artery disease - Juntendo University trial: effects of miglitol on endothelial vascular reactivity in type 2 diabetic patients with coronary heart disease (J-MACH). Circ J.

[15] Basu A, Charkoudian N, Schrage W (2007). Beneficial effects of GLP-1 on endothelial function in humans: dampening by glyburide but not by glimepiride. Am J Physiol Endocrinol Metab.

[16] Ceriello A, Esposito K, Testa R (2011). The possible protective role of glucagon-like peptide 1 on endothelium during the meal and evidence for an ‘endothelial resistance’ to glucagon-like peptide 1 in diabetes. Diabetes Care.

[17] Nyström T, Gutniak MK, Zhang Q (2004). Effects of glucagon-like peptide-1 on endothelial function in type 2 diabetes patients with stable coronary artery disease. Am J Physiol Endocrinol Metab.

[18] Hattori Y, Jojima T, Tomizawa A (2010). A glucagon-like peptide-1 (GLP-1) analogue, liraglutide, upregulates nitric oxide production and exerts anti-inflammatory action in endothelial cells. Diabetologia.

[19] Qin X, Shen H, Liu M (2005). GLP-1 reduces intestinal lymph flow, triglyceride absorption, and apolipoprotein production in rats. Am J Physiol Gastrointest Liver Physiol.

[20] Wøjdemann M, Wettergren A, Sternby B (1998). Inhibition of human gastric lipase secretion by glucagon-like peptide-1. Dig Dis Sci.

[21] Hsieh J, Longuet C, Baker CL (2010). The glucagon-like peptide 1 receptor is essential for postprandial lipoprotein synthesis and secretion in hamsters and mice. Diabetologia.

[22] Kato T, Inoue T, Node K (2010). Postprandial endothelial dysfunction in subjects with new-onset type 2 diabetes: an acarbose and nateglinide comparative study. Cardiovasc Diabetol.

[23] Kitano D, Chiku M, Li Y (2013). Miglitol improves postprandial endothelial dysfunction in patients with acute coronary syndrome and new-onset postprandial hyperglycemia. Cardiovasc Diabetol.

[24] Masuda D, Nakagawa-Toyama Y, Nakatani K (2009). Ezetimibe improves postprandial hyperlipidaemia in patients with type IIb hyperlipidaemia. Eur J Clin Invest.

